# NGS-based Molecular diagnosis of 105 eyeGENE^®^ probands with Retinitis Pigmentosa

**DOI:** 10.1038/srep18287

**Published:** 2015-12-15

**Authors:** Zhongqi Ge, Kristen Bowles, Kerry Goetz, Hendrik P. N. Scholl, Feng Wang, Xinjing Wang, Shan Xu, Keqing Wang, Hui Wang, Rui Chen

**Affiliations:** 1 Human Genome Sequencing Center, Baylor College of Medicine, Houston, Texas, USA; 2 Department of Molecular and Human Genetics, Baylor College of Medicine, Houston, Texas, USA; 3 Ophthalmic Genetics and Visual Function Branch, National Eye Institute/National Institutes of Health, Bethesda, Maryland, USA; 4 Wilmer Eye Institute, Johns Hopkins University, Baltimore, Maryland, USA; 5 Program in Developmental Biology, Baylor College of Medicine, Houston, Texas, USA; 6 The Verna and Marrs Mclean Department of Biochemistry and Molecular Biology, Baylor College of Medicine, Houston, Texas, USA; 7 Structural and Computational Biology and molecular Biophysics Graduate Program, Baylor College of Medicine, Houston, Texas, USA

## Abstract

The National Ophthalmic Disease Genotyping and Phenotyping Network (eyeGENE^®^) was established in an effort to facilitate basic and clinical research of human inherited eye disease. In order to provide high quality genetic testing to eyeGENE^®^’s enrolled patients which potentially aids clinical diagnosis and disease treatment, we carried out a pilot study and performed Next-generation sequencing (NGS) based molecular diagnosis for 105 Retinitis Pigmentosa (RP) patients randomly selected from the network. A custom capture panel was designed, which incorporated 195 known retinal disease genes, including 61 known RP genes. As a result, disease-causing mutations were identified in 52 out of 105 probands (solving rate of 49.5%). A total of 82 mutations were identified, and 48 of them were novel. Interestingly, for three probands the molecular diagnosis was inconsistent with the initial clinical diagnosis, while for five probands the molecular information suggested a different inheritance model other than that assigned by the physician. In conclusion, our study demonstrated that NGS target sequencing is efficient and sufficiently precise for molecular diagnosis of a highly heterogeneous patient cohort from eyeGENE^®^.

Retinitis Pigmentosa (RP) is the most common form of inherited retinal degeneration, which has an estimated prevalence of 1 in 3,500–4,000 individuals^1^. RP patients first experience night blindness, followed by impaired daytime vision with visual fields gradually reduced from mid-periphery to the center due to the degeneration of rod photoreceptors followed by cone photoreceptors over time^2^. RP is genetically heterogeneous and more than 60 genes have been linked to the disease (RetNet)^3^. Molecular diagnosis is particularly challenging for RP patients for two reasons. First, the inheritance pattern is complex, including autosomal dominant, autosomal recessive, X-linked, and digenic as well as mitochondrial inherited forms^4^. More than half of RP cases are simplex, where the mode of inheritance is unclear, making the interpretation of variants more challenging. Second, there is extensive phenotypic and genetic overlap between RP and other retinal diseases or syndromic diseases with an eye phenotype, making it necessary to screen mutations in a large number of genes, not just those strictly associated with RP^5^,^6^.

The traditional diagnostic test for genetic and allelic heterogeneous diseases such as RP has been limited to Sanger sequencing and arrayed primer extension (APEX)^7^. However, Sanger sequencing, while accurate, is prohibitively costly and time consuming for large-scale screening. On the other hand, APEX can only detect known mutations, resulting in a low diagnostic yield for genetically heterogeneous conditions like RP. For example, a typical genetic diagnostic rate for autosomal recessive RP using APEX is reported to be 10% in a recent study^8^. In comparison, Next-generation sequencing (NGS) based technology, which allows multiple genes to be sequenced at the same time, has emerged as a robust, cost effective and accurate method. Recent studies utilizing NGS based method for molecular diagnosis of RP achieved a diagnostic rate ranging from 36% to 60%^9^,^10^,^11^. As a result, NGS based genetic testing is gradually being adopted as the method of choice for molecular diagnosis of RP patients, an important step towards better clinical diagnosis, prognosis, and identifying patients who may benefit from therapeutic interventions such as gene therapy^10^,^12^,^13^.

The National Ophthalmic Disease Genotyping and Phenotyping Network (eyeGENE^®^) is a multicenter genomic medicine initiative started by the National Eye Institute (NEI) at the National Institutes of Health (NIH) in 2006. eyeGENE^®^ aims to promote studies of inherited eye diseases and their genetic causes. The program includes a CLIA-level DNA repository, a database linking genotype and phenotype data, and a patient registry. eyeGENE^®^ not only expands patients’ access to diagnostic testing, but also allows registered researchers to gain access to the research database and samples for continued studies such as genotype-phenotype correlations, disease causing mutation prevalence and novel disease gene discovery^14^,^15^. Additionally, eyeGENE^®^ is able to contact specific patient populations from the registry for recruitment of additional clinical studies.

The largest patient population in eyeGENE^®^ is RP with total over 2,000 patients, of which about 70% are simplex cases. Due to the high cost and complexity of molecular diagnosis of RP, the vast majority of the simplex RP cases and some of the multiplex cases had not been tested, representing one of the biggest challenges for fulfilling eyeGENE^®^’s mission. To address this issue, we conducted a pilot study and performed NGS based mutation screening of 105 RP probands from eyeGENE^®^ whose molecular diagnosis remained unknown. This cohort was tested using a custom designed 195-gene panel, which included 61 known RP causative genes and 19 genes that cause syndromic RP such as Usher and Bardet-Biedl syndrome. Through NGS based sequencing analysis we assigned causative mutations to 52 patients, achieving a solving rate of 49.5%. While 49 patients carried mutations in known RP genes, 3 patients were found to carry mutations in retinal disease genes that have not been associated with nonsyndromic RP previously. In addition, 5 of the 6 RP families initially labeled as autosomal dominant were found to carry compound heterozygous or homozygous mutations in known RP disease genes. Taken together, our results indicate that an NGS based approach is effective in providing a diagnosis for the highly heterogeneous patient collection at eyeGENE^®^.

## Materials and Methods

### Clinical identification of RP patients

Patients with inherited eye disease were enrolled in the eyeGENE^®^ program (protocol #06-EI-0236) by approved certified eye care specialist. Clinical details and family history were provided by referring clinicians and entered into the eyeGENE^®^ database (https://nationaleyegene.nei.nih.gov/eyeGENE). Clinical information and family history were further reviewed by members of the eyeGENE^®^ Working Group to corroborate the patient’s diagnosis of RP. For this study, 105 unrelated RP probands were randomly selected from the eyeGENE^®^ database. Informed consent was obtained from tested individuals or from parents or guardians for individuals under age 18. All experimental protocols were approved by the Institutional Review Board of Baylor College of Medicine. This study adhered to the Declaration of Helsinki.

### DNA extraction, library preparation and capture sequencing

For each patient enrolled in eyeGENE^®^, a blood sample was collected and shipped to the eyeGENE^®^ Coordinating Center CLIA (Clinical Laboratory Improvement Amendments) laboratory on the NIH campus in Bethesda, MD. Genomic DNA was extracted from whole blood either manually or automatically using the Gentra Puregene (Qiagen). DNA concentration was measured by a NanoDrop 1000 spectrophotometer (Thermo Scientific, Wilmington, DE) and samples were stored indefinitely in the eyeGENE^®^ Biobank at NEI. A fraction of de-identified DNA was send to Baylor College of Medicine for diagnostic research testing. Genomic DNA from each sample was mechanically sheared, end repaired, and ligated to molecularly bar-coded adaptors to generate sequencing libraries following the manufacturer’s standard protocol (Illumina). Co-capture was performed on pooled DNA libraries in groups of up to 48 samples. Captured sample DNA was sequenced on an Illumina HiSeq 2000 according to the standard operating protocol.

### Capture panel design

A capture panel enriched of the retinal disease genes was developed and assessed as described previously^16^. The panel covers coding exons and flanking splicing junctions for 195 known retinal disease genes at the time of design (Supplemental Table 1). A total of 61 nonsyndromic RP associated genes were included in the panel including 18 adRP genes and 33 arRP genes, 3 X-linked RP genes, and 7 RP genes that can be both dominant and recessive (Supplementary Table S1).

### Bioinformatics analysis

An automated pipeline previously described was used to process sequencing data with reads mapping, recalibration, realignment, variant calling, variant filtering and annotation^17^. Since RP is a rare Mendelian disease, recessive variants with an allele frequency >0.5% or dominant variants with an allele frequency >0.1% in the following databases were filtered out: the 1000 genome database^18^, dbSNP135 (National Center for Biotechnology Information, http://www.ncbi.nlm.nih.gov/SNP/), the NHLBI Exome Sequencing database (http://evs.gs.washington.edu/EVS/), the NIEHS Exome Sequencing database (http://evs.gs.washington.edu/niehsExome/), as well as an internal control database of 997 exomes. The pathogenicity of these rare variants was assessed based on three criteria. First of all, variants reported in the Human Gene Mutation Database (HGMD)^19^ or the primary literature were identified. Secondly, variants that lead to severe loss of function mutations such as stopgain, stoploss, frameshift and splicing defects were identified. Third, missense variants that result in protein coding changes were evaluated by the *in silico* prediction program dbNSFP^20^ and only deleterious ones predicted by 3 out of the 6 algorithms (SIFT, Polyphen2, LRT, MutationTaster, MutationAssessor, and PhyloP) were considered as candidates.

### Validation and Report

All putative causative mutations identified were validated by Sanger sequencing. A 500-bp flanking sequence at each side of the mutation was obtained from the UCSC genome browser. RepeatMasker was used to mask the repetitive region. Primer 3 was used to design a pair of primers at least 50 bp upstream and downstream from the mutation. After PCR amplification, the amplicons were sequenced on an ABI 3730xl or 3500XL Genetic Analyzer. Reports of the high-confidence genetic testing results were sent back to eyeGENE^®^ and positive results were confirmed through direct sequencing by the CLIA certified laboratories in the eyeGENE^®^ Network. Confirmed results were then shared with the referring clinician.

## Results

### The RP patient cohort

**Figure 1:**
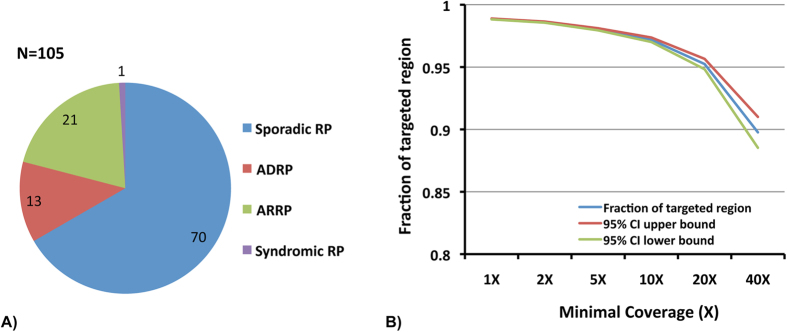
The model of inheritance of the 105 RP probands and the sequencing quality. (**A**) Majority of the 105 RP probands were simplex or unknown. (**B**) Fractions of targeted region with minimal coverage from 1X to 40X showed high quality sequencing results.

A total of 105 unrelated RP probands whose mutations remained unknown were randomly selected from the eyeGENE® database. Based on inheritance information documented in the database, most of the cases were simplex or unknown (67%), followed by autosomal recessive (20%), and autosomal dominant (13%). There was also one case of syndromic RP with hearing loss (Fig. 1a). The majority of the probands is Caucasian (65%), followed by unknown descent (19%), Asian (9%), African American (4%), and multiple races (2%). Among them, sixteen of the probands had been screened for mutations using Sanger direct sequencing in a subset of known RP genes, including *ABCA4*, *CDH23*, *CLRN1*, *DFNB31*, *IMPDH1*, *KLHL7*, *NR2E3*, *PCDH15*, *PRPF3*, *PRPF8*, *PRPF31*, *RDS*, *RHO*, *RP1, RP2*, *RPGR*, *TOPORS*, *USH1C*, *USH1G*, and *USH2A* (Supplementary Table S2).

### Identification of pathogenic mutations

To identify the pathogenic mutations in the 105 RP patients, NGS based panel sequencing that covers all coding exons and flanking splicing junctions of 195 known retinal disease genes was performed. Ten and twenty fold average coverage was achieved for 97% and 95% of the targeted regions, respectively (Fig. 1b). Sequencing results were analyzed using the bioinformatics pipeline as described in the method section. Known mutations were identified by searching the public databases, such as the HGMD database^19^, while novel variants were annotated for their impact on protein coding. As a result, putative mutations were found in 52 cases with a solving rate of 49.5% (Table 1).

**Figure 2:**
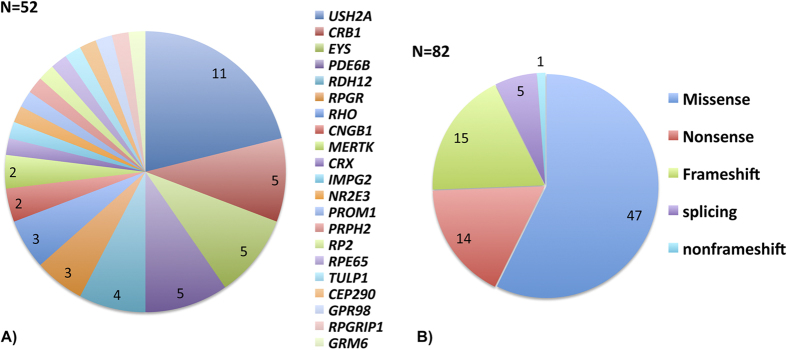
Disease-causing mutations were found for 52 probands and the majority of the mutations were missense. (**A**) 21 retinal disease genes were assigned causal in the 52 solved cases. (**B**) A total of 82 mutations were identified along with their different types.

The mutations are distributed across 21 retinal disease genes with *USH2A* as the most frequently mutated gene, accounting for 11 solved cases (22%). In addition, mutations have been found in *EYS* (10%), *CRB1* (10%), *PDE6B* (10%)*, RDH12* (8%), *RPGR* (6%)*, RHO* (6%), *CNGB1* (4%), *MERTK* (4%), and the rest of cases in 12 genes with one case for each gene (Fig. 2a). A total of 82 mutant alleles have been identified in our study, all of which have been confirmed by the CLIA certified laboratories in the eyeGENE^®^ Network. Among them, missense mutations account for 58%, followed by frameshift (18%), nonsense (17%), and splicing (7%) (Fig. 2b). Interestingly, close to 60% of the mutant alleles have not been previously reported (48/82).

#### Identification of pathogenic mutations in dominant and recessive cases

**Figure 3:**
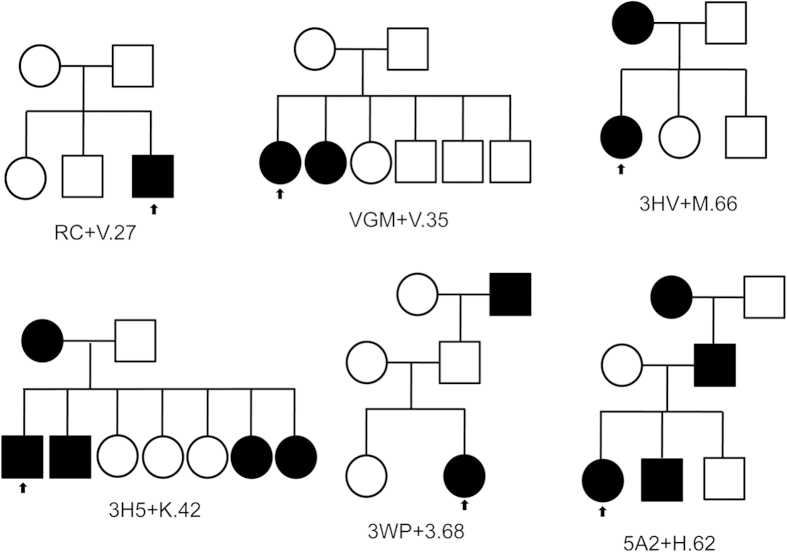
Pedigrees for 6 probands labeled dominant inheritance in the eyeGENE^®^ database. The small arrow indicates the proband sequenced in each family.

As shown in Fig. 1, a total of 13 RP cases were labeled as dominant inheritance based on the information documented in the eyeGENE^®^ database. Among them, mutations were identified for 6 patients (Table 1). Consistent with the dominant inheritance model, a previously reported dominant mutation was found in *RHO* for one patient 5A2+H.62. In addition, homozygous or compound heterozygous mutations in recessive RP disease genes including *USH2A*, *EYS*, *MERTK* and *PDE6B* were identified for patients RC+V27, VGM+V.35, 3HV+M.66 and 3H5+K.42, respectively. Finally, for patient 3WP+3.68, a causal homozygous mutation was identified in *RDH12*, which can either cause recessive or dominant RP (Table 1). These patients have been assigned with a dominant inheritance model based on the initial diagnosis provided by the physician. Our molecular diagnosis results did not support this except for patient 5A2+H.62, so we contacted the clinicians for further information. Investigation of the pedigree information indicated that indeed some of the families were likely to be misclassified (Fig. 3). For example, in both RC+V.27 and VGM+V.35 families all affected individuals appear in the same generation while their parents are normal, suggesting that the inheritance model for these two families is indeed recessive. For the other three families, evidence of a dominant inheritance model is weak, because the pedigrees either lack male to male transmission (3WP+3.68), or only show affected members in 2 generations (3HV+M.66 and 3H5+K.42).

There were total 21 patients labeled as recessive inheritance and 9 of them were solved (Table 1). Consistent with the diagnosis of arRP, all genes found were known arRP genes, including *CRB1*, *PDE6B*, *RDH12*, *RPE65*, and *USH2A*. Of the solved arRP cases, one interesting case was the c.295C>A, p.(Ile2995Asn) missense mutation in homozygous state in *RDH12* found in proband 3JY+V.17, which is also the causal mutation for the proband 3WP+3.68 (Table 1). This missense mutation leads to LCA^21^ when combined with a nonsense mutation, while severe RP^22^ is observed when combined with a second missense allele. Consistent with the idea that the c.295C>A, p.(Ile2995Asn) is a hypomorphic allele, both probands 3WP+3.68 and 3JY+V.17 show RP phenotype at age 11 and 3 years old, respectively. Therefore, both cases turned out to have relatively earlier onset age compared to typical RP patients and should be classified as juvenile RP. This is also in consistent with a recent research shown that *RDH12* was the most frequently mutated gene in the juvenile RP group in a large Spanish cohort^8^.

#### Identification of pathogenic mutations in simplex/unknown cases

In this study, 70 (67%) of the RP cases were simplex or unknown, for which molecular diagnosis is most valuable. In the 70 simplex cases, causative mutations were identified in 37 samples. Specifically, we identified mutations in autosomal dominant retinal disease genes for 5 cases, in X-linked disease genes for 4 cases, and in recessive disease genes for 28 cases.

Dominant mutations in RP genes *PROM1*, *PRPH2*, *RHO*, and *CRX* were found in 5 probands (Table 1). While one novel nonsense mutation in *CRX* was found in patient 3XM+J.87, four mutations previously reported were found in genes *PRPH2*, *PROM1*, and *RHO*. For example, the p.(Arg172Trp) mutation in *PRPH2* was assigned causative mutation for proband 8J+Y.4. In a previous study, the p.(Arg172Trp) mutation in *PRPH2* was found to segregate in two independent families with affected members showing symptoms with blurred central vision and photophobia, while no complain of night blindness or restricted peripheral vision^23^. A closer investigation of the clinical exam result for proband 8J+Y.4 showed that this patient had both macular and peripheral retinal degeneration and that he experienced visual acuity loss (at 24 years) before night blindness (at 46 years). This is similar to the phenotype described in the previous study supporting that p.(Arg172Trp) in *PRPH2* is likely the causative mutation. For another example, proband N6+A.15 was assigned the p.(Arg373Cys) mutation in *PROM1*. This mutation has been reported in a four-generation Italian family with autosomal dominant RP and affected members showing reduced central vision first and with night blindness progressing over time^24^. In proband N6+A.15, however, night blindness and visual acuity loss occurred at the same time (at age 30 years). It is possible that genetic background or environment factors could influence the onset of development of night blindness in patients with the p.(Arg373Cys) mutation in *PROM1*. Finally, p.(Ala164Val) and p.(Pro171Leu) mutations in *RHO* were found in patients S7+G.76 and 5VY+V.14, respectively. Both have been previously reported to be causal mutations and segregate in dominant RP families^25^,^26^, and both affect folding of rhodopsin protein by biochemistry studies^27^.

X-linked RP is estimated to account for 10% ~ 20% of all RP, of which the males typically show an early age of onset and a rapid course of vision loss. *RPGR* and *RP2,* the genes most often associated with X-linked RP, explain more than 15% of isolated male RP cases^28^. In this study, we identified hemizygous mutations in *RPGR* in 3 probands and *RP2* in 1 proband (Table 1). Two out of the three *RPGR* mutations, p.(Glu749*) and p.(Glu1014Glyfs*64), were novel and were located at the mutation hot spot *RPGR* ORF15. All three mutations are likely to be loss of function mutations that either result in truncated proteins or no protein through nonsense mediated decay. The p.(Leu240Tyrfs*14) identified in *RP2* was also novel and predicted to produce a prematurely truncated protein.

For 28 of the simplex/unknown cases, mutations were identified in 10 arRP genes (Table 1). As expected, the most frequently mutated genes were *USH2A* and *EYS*, accounting for 8 and 4 cases, respectively. Additionally, for three probands we found deleterious mutations in retinal disease genes other than those associated with RP (Table 1). For example, compound heterozygous mutations c.4349A>G, p.(Lys1450Arg) and c.2285G>A , p.(Arg762His) that are novel and predicted to be damaging, were found in Usher gene *GPR98* in patient 5ES+3.87. Although the age of diagnosis of hearing loss in type II Usher patients can be variable, it is generally during childhood with a median age of 5 years^29^. However, patient 5ES+3.87 did not show any hearing loss at the time of the clinical visit when he was 39 years old, it is thus unlikely that patient 5ES+3.87 could be a typical type II Usher patient (Table 2). In another proband UFC+7.74, novel missense mutations p.(Val243Phe) and p.(Ser747Leu), predicted to be damaging, were found in the complete type of Congenital Stationary Night-blindness (CSNB) gene *GRM6*. Electroretinogram (ERG) responses for this patient were not recordable in either eye under scotopic and photopic conditions (Table 2 and supplementary Fig. S2). Finally, compound heterozygous mutations p.(Phe1950Leufs*15) and p.(Glu1803Asp) in *CEP290* were found in proband 5WY+Y.91. The first allele, p.(Phe1950Leufs*15) in *CEP290* has been previously reported in two LCA families in compound heterozygous state with either a splicing mutation or a non-frameshift mutation, and the second allele p.(Glu1803Asp) was novel^30^. A closer investigation of the clinical information of this patient showed that she had first experienced night-blindness at age 18 and vision loss at age 22. Also, her best corrected visual acuity was 20/20 in both eyes at the time of clinical visit when she was 39 years of age.

## Discussion

One of the biggest challenges for fulfilling eyeGENE^®^’s mission is that most of its enrolled patients were not introduced to a clear plan of genetic testing that would be both financially efficient and result in a likely associated genetic cause. Of these patients, the majority (70%) is RP, which is highly heterogeneous at multiple levels. First, RP is both genetically and clinically heterogeneous with multiple genes contributing to the disease, and phenotypes vary greatly among patients. Second, the inheritance pattern is heterogeneous and not always clear based on the pedigree information alone. Finally, as a national network, the eyeGENE^®^ patients were recruited and examined by many physician groups across the country thus the clinical tests performed and the diagnosis criteria are not always the same. The information for each proband available also varies significantly. As a result, molecular diagnosis of this highly heterogeneous collection is challenging. Here, we performed a pilot study using NGS based panel sequencing for molecular diagnosis of eyeGENE^®^ RP patients and achieved a similar yield of solved cases (~50%) in comparison to previous studies that adopted similar approaches^10^,^11^. In addition, of the 82 mutations identified, 48 (~60%) of them were novel, which is also comparable to previous studies^10^,^11^.

One interesting finding of this study is the inconsistency between inheritance patterns assigned and the genetic test results for five out of six adRP solved cases (Table 1 and Fig. 3). While two of the families were misclassified (RC+V.27 and VGM+V.35), the other three families are inconclusive for dominant inheritance as members from less than three generations were affected. Indeed, assigning inheritance patterns based solely on pedigree information could be prone to error. For example, 8.5% of the families thought to have adRP truly have X-linked RP^31^. Using the NGS approach, however, helps to clarify this issue since all variants, regardless of inheritance, are considered simultaneously.

Our study also showed that NGS based molecular diagnosis can potentially reveal novel genotype phenotype associations. For example, in three cases, we identified potential new associations for mutations in retinal disease genes *GPR98*, *CEP290* and *GRM6* with an RP phenotype. Although the documented clinical information for these three patients supported RP phenotype, it is possible that these patients had Usher syndrome, LCA, or CSNB and were misdiagnosed as general RP. Segregation tests as well as clinical diagnosis refinements will be required to confirm the genetic testing results. Nevertheless, these findings are particularly important for the family, especially family members at risk. With the identification of many more mutations causing inherited retinal diseases and their associated phenotype clearly documented in the eyeGENE^®^ database, clinicians and counselors will feel more confident in providing guidance to affected families.

In conclusion, our study showed that NGS based approach is robust and effective in providing precise molecular diagnosis for the highly heterogeneous collection of RP patients from eyeGENE^®^. The results from this study are essential for fulfilling the goals of eyeGENE^®^ to advance vision research and to contribute to the shared resources for the research community. First, the novel mutations identified in these RP patients will be documented in the database and accessible to other research groups to continue the research cycle, which will provide valuable information for genotype-phenotype correlation studies in the future. Secondly, patient samples without assigned mutations represent a valuable resource for novel RP gene discovery. In fact, novel RP genes have been identified from these samples and have lead to existing publications^32^. Last but not least, genetic testing results will provide registered eyeGENE^®^ patients the information and opportunity to participate in gene-specific clinical trials.

## Additional Information

**How to cite this article**: Ge, Z. *et al.* NGS-based Molecular diagnosis of 105 eyeGENE® probands with Retinitis Pigmentosa. *Sci. Rep.* **5**, 18287; doi: 10.1038/srep18287 (2015).

## Supplementary Material

Supplementary Information

## Figures and Tables

**Table 1:** Pathogenic mutations were identified in 52 patients

ID	Gene	NM ID	Genotype	cDNA change	Protein change	References
ADRP						
VGM+V.35	*EYS*	NM_001142800	Heterozygous	c.8984T>A	p.(Ile2995Asn)	PMID: 20537394^33^
			Heterozygous	c.7095T>G	p.(Tyr2365*)	PMID: 20537394^33^
3HV+M.66	*MERTK*	NM_006343	Homozygous	c.1787-2A>C	p.?	Novel
3H5+K.42	*PDE6B*	NM_000283	Heterozygous	c.173C>T	p.(Ala58Val)	Novel
			Heterozygous	c.2401C>T	p.(Gln801*)	Novel
3WP+3.68	*RDH12*	NM_152443	Homozygous	c.295C>A	p.(Leu99Ile)	PMID: 15322982^21^
5A2+H.62	*RHO*	NM_000539	Heterozygous	c.1040C>T	p.(Pro347Leu)	PMID: 2215617^34^
RC+V.27	*USH2A*	NM_206933	Heterozygous	c.9815C>T	p.(Pro3272Leu)	PMID: 18281613^35^
			Heterozygous	c.10342G>A	p.(Glu3448Lys)	PMID: 24265693^36^
ARRP						
VGJ+4.64	*CRB1*	NM_201253	Homozygous	c.2401A>T	p.(Lys801*)	PMID: 11389483^37^
3UF+P.83	*CRB1*	NM_201253	Homozygous	c.3961T>C	p.(Cys1321Arg)	Novel
5WL+S.22	*CRB1*	NM_201253	Heterozygous	c.3997G>A	p.(Glu1333Lys)	Novel
			Heterozygous	c.3853T>C	p.(Cys1285Arg)	Novel
59H+2.32	*PDE6B*	NM_000283	Heterozygous	c.2116A>T	p.(Lys706*)	PMID: 7724547^38^
			Heterozygous	c.292C>T	p.(Arg98Cys)	Novel
			Heterozygous	c.2093_2094insCCTGT	p.(Leu701Cysfs*14)	Novel
3JY+V.17	*RDH12*	NM_152443	Homozygous	c.295C>A	p.(Leu99Ile)	PMID: 15322982^21^
57R+R.78	*RDH12*	NM_152443	Homozygous	c.377C>T	p.(Ala126Val)	PMID: 19140180^39^
347+7.8	*RPE65*	NM_000329	Heterozygous	c.310G>A	p.(Gly104Ser)	Novel
			Heterozygous	c.432C>G	p.(Tyr144*)	Novel
			Heterozygous	c.2299delG	p.(Glu767Serfs*21)	PMID: 9624053^40^
U92+K.87	*USH2A*	NM_206933	Heterozygous	c.4714C>T	p.(Leu1572Phe)	PMID: 22025579^41^
			Heterozygous	c.11105G>A	p.(Trp3702*)	PMID: 23591405^9^
JX+6.76	*USH2A*	NM_206933	Homozygous	c.5012G>A	p.(Gly1671Asp)	Novel
Simplex/unknown RP						
5WY+Y.91	*CEP290*	NM_025114	Heterozygous	c.5409A>C	p.(Glu1803Asp)	Novel
			Heterozygous	c.5850delT	p.(Phe1950Leufs*15)	PMID: 17345604^30^
8G+Y.78	*CNGB1*	NM_001297	Homozygous	c.3150delG	p.(Phe1051Leufs*12)	PMID: 24043777^42^
3XC+7.8	*CNGB1*	NM_001297	Heterozygous	c.2805delG	p.(Glu935Aspfs*2)	Novel
			Heterozygous	c.2544_2545insG	p.(Leu849Alafs*3)	Novel
U7U+9.12	*CRB1*	NM_201253	Homozygous	c.2501G>A	p.(Gly834Asp)	Novel
UEW+W.58	*CRB1*	NM_201253	Heterozygous	c.3712T>C	p.(Cys1238Arg)	Novel
			Heterozygous	c.252_253insTG	p.(Asn87*)	Novel
3XM+J.87	*CRX*	NM_000554	Heterozygous	c.682C>T	p.(Gln228*)	Novel
U6H+2.34	*EYS*	NM_001142800	Heterozygous	c.6078G>T	p.(Gln2026His)	Novel
			Heterozygous	c.6416G>A	p.(Cys2139Tyr)	PMID: 20333770^43^
TW+H.97	*EYS*	NM_001142800	Heterozygous	c.4350_4356del	p.(Ile1451Profs*3)	PMID: 20537394^33^
			Heterozygous	c.6714delT	p.(Ile2239Serfs*17)	PMID: 18976725^44^
3U6+9.42	*EYS*	NM_001142800	Heterozygous	c.904C>T	p.(Leu302Phe)	Novel
			Heterozygous	c.8860T>C	p.(Phe2954Leu)	Novel
			Homozygous	c.3250A>C	p.(Thr1084Pro)	Novel
VNM+T.47	*EYS*	NM_001142800	Homozygous	c.4402G>C	p.(Asp1468His)	Novel
			Homozygous	c.3443+1G>T	p.?	Novel
5ES+3.87	*GPR98*	NM_032119	Heterozygous	c.2285G>A	p.(Arg762His)	Novel
			Heterozygous	c.4349A>G	p.(Lys1450Arg)	Novel
UFC+7.74	*GRM6*	NM_000843	Heterozygous	c.727G>T	p.(Val243Phe)	Novel
			Heterozygous	c.2240C>T	p.(Ser747Leu)	Novel
3XN+K.89	*IMPG2*	NM_016247	Heterozygous	c.1589C>A	p.(Ser530*)	Novel
			Heterozygous	c.3030_3031insTTTTAGGTGATGAA	p.(Ala1011Phefs*2)	Novel
5VR+W.92	*MERTK*	NM_006343	Heterozygous	c.390G>A	p.(Trp130*)	PMID: 24154662^10^
			Heterozygous	c.2287C>A	p.(Pro763Thr)	Novel
3V5+8.13	*NR2E3*	NM_014249	Heterozygous	c.995-2A>C	p.?	Novel
			Heterozygous	c.226C>T	p.(Arg76Trp)	PMID: 10655056^45^
3U3+6.63	*PDE6B*	NM_000283	Heterozygous	c.2193+1G>A	p.?	PMID: 7724547^38^
			Heterozygous	c.299G>A	p.(Arg100His)	PMID: 22334370^46^
UGQ+Q.72	*PDE6B*	NM_000283	Heterozygous	c.892C>T	p.(Gln298*)	PMID: 8394174^47^
			Heterozygous	c.2116A>T	p.(Lys706*)	PMID: 7724547^38^
MK+W.33	*PDE6B*	NM_000283	Homozygous	c.1540delC	p.(Leu514Trpfs*61)	Novel
N6+A.15	*PROM1*	NM_006017	Heterozygous	c.1117C>T	p.(Arg373Cys)	PMID: 20393116^24^
8J+Y.4	*PRPH2*	NM_000322	Heterozygous	c.514C>T	p.(Arg172Trp)	PMID: 8485576^23^
34U+F.88	*RDH12*	NM_152443	Homozygous	c.805_809del	p.(Ala269Glyfs*2)	Novel
S7+G.76	*RHO*	NM_000539	Heterozygous	c.491C>T	p.(Ala164Val)	PMID: 7981701^25^
5VY+V.14	*RHO*	NM_000539	Heterozygous	c.512C>T	p.(Pro171Leu)	PMID: 1833777^26^
U6Z+5.73	*RP2*	NM_006915	Hemizygous	c.718delT	p.(Leu240Tyrfs*14)	Novel
9C+Y.10	*RPGR*	NM_001034853	Hemizygous	c.2245G>T	p.(Glu749*)	Novel
U2C+J.77	*RPGR*	NM_001034853	Hemizygous	c.3039_3040del	p.(Glu1014Glyfs*64)	PMID: 23681342^48^
UNM+T.54	*RPGR*	NM_000328	Hemizygous	c.1495_1496insA	p.(Ile499Asnfs*14)	Novel
59R+5.99	*RPGRIP1*	NM_020366	Heterozygous	c.1753C>T	p.(Pro585Ser)	PMID: 21153841^49^
			Heterozygous	c.2302C>T	p.(Arg768*)	PMID: 20079931^50^
			Heterozygous	c.973T>C	p.(Phe325Leu)	Novel
5FP+L.15	*TULP1*	NM_003322	Heterozygous	c.1213G>C	p.(Ala405Pro)	Novel
			Heterozygous	c.1495C>T	p.(Pro499Ser)	Novel
5FV+T.56	*USH2A*	NM_206933	Heterozygous	c.9921T>G	p.(Cys3307Trp)	PMID: 21569298^51^
			Heterozygous	c.13010C>T	p.(Thr4337Met)	PMID: 20507924^52^
SS+6.62	*USH2A*	NM_206933	Heterozygous	c.2276G>T	p.(Cys759Phe)	PMID: 10775529^53^
			Heterozygous	c.10073G>A	p.(Cys3358Tyr)	PMID: 20507924^52^
32V+Y.3	*USH2A*	NM_206933	Heterozygous	c.842C>A	p.(Thr281Lys)	PMID: 22135276^54^
			Heterozygous	c.6795_6797del	p.(Glu2265_Tyr2266delinsAsp)	PMID: 18273898^55^
P9+A.52	*USH2A*	NM_206933	Heterozygous	c.6172_6173insA	p.(Val2059Glyfs*44)	Novel
			Heterozygous	c.2276G>T	p.(Cys759Phe)	PMID: 10775529^53^
5ZU+U.41	*USH2A*	NM_206933	Homozygous	c.5012G>A	p.(Gly1671Asp)	Novel
VHM+Y.45	*USH2A*	NM_206933	Heterozygous	c.5167G>C	p.(Gly1723Arg)	Novel
			Heterozygous	c.4370C>A	p.(Ser1457*)	Novel
				c.14792-2A>G	p.?	PMID: 22025579^41^
8X+A.29	*USH2A*	NM_206933	Heterozygous	c.6779C>A	p.(Ser2260Tyr)	Novel
			Heterozygous	c.12094G>A	p.(Gly4032Arg)	Novel
			Heterozygous	c.2299delG	p.(Glu767Serfs*21)	PMID: 9624053^40^
5CV+J.77	*USH2A*	NM_206933	Heterozygous	c.4714C>T	p.(Leu1572Phe)	PMID: 22025579^41^
			Heterozygous	c.9433C>T	p.(Leu3145Phe)	Novel

**Table 2:** Clinical information for 3 probands in which mutations in other retinal disease genes not previously associated with non-syndromic RP were found.

ID	Gene	Disease previously associated	Patient Clinical Phenotype							
			Age (years) when patient first aware of		Best corrected visual acuity	Hearing defects	Electroretinogram (Amplitude μV, Implicit time ms)			
			Night blindness	Vision loss			Dark-adapted		Light-adapted	
							OD	OS	OD	OS
5WY+Y.91	*CEP290*	Leber congenital amaurosis	18	22	OD 20/20 OS 20/20	No	12, 33	16, 35	11, 37	12, 36
5ES+3.87	*GPR98*	Usher syndrome	37	37	OD 20/20 OS 20/20	No	43, 12	40, 24	30, 37	30, 38
UFC+7.74	*GRM6*	Congenital stationary night blindness	25	25	OD 20/20 OS 20/25	No	NR	NR	NR	NR

All three patients underwent electroretinogram (ERG) tests following the ISCEV (International Society for Clinical Electrophysiology of Vision) standard. NR: not recordable
