# Supplementary material for: NGS-based Molecular diagnosis of 105 eyeGENE® probands with Retinitis Pigmentosa
Source: Sci Rep. 2015 Dec 15;5:18287. doi: 10.1038/srep18287 (PMC4678898; doi:10.1038/srep18287)
Supplement: Supplementary Information [file srep18287-s1.pdf]

Supplementary Information

NGS-based Molecular diagnosis of 105 eyeGENE® probands with Retinitis Pigmentosa

Zhongqi Ge[1,2], Kristen Bowles[4], Kerry Goetz[3], Hendrik P. N. Scholl[4], Feng Wang[1,2], Xinjing Wang[3], Shan Xu[1], Keqing Wang[2], Hui Wang[1,2], Rui Chen[1,2,5,6,7*]

[1]*Human Genome Sequencing Center, Baylor College of Medicine, Houston, Texas, USA*

[2]*Department of Molecular and Human Genetics, Baylor College of Medicine, Houston, Texas, USA*

[3]*Ophthalmic Genetics and Visual Function Branch, National Eye Institute/National Institutes of Health, Bethesda, Maryland, USA*

[4]*Wilmer Eye Institute, Johns Hopkins University, Baltimore, Maryland, USA*

5 Program in Developmental Biology, Baylor College of Medicine, Houston, Texas, USA

6 The Verna and Marrs Mclean Department of Biochemistry and Molecular Biology, Baylor College of Medicine, Houston, Texas, USA

7 Structural and Computational Biology and molecular Biophysics Graduate Program, Baylor College of Medicine, Houston, Texas, USA

Correspondence: Rui Chen, 1 Baylor Plaza, Room N1519, Baylor College of Medicine, Houston, TX 77030, USA

ruichen@bcm.edu

Supplemental Table 1 Genes in our retinal capture panel

All genes in the panel	RP genes in the panel	Inheritance mode of RP genes
ABCA4	*ABCA4*	recessive
ABCC6	*BBS1*	recessive
ABHD12	*BEST1*	recessive/dominant
ADAM9	*C2orf71*	recessive
TREX1	*C8orf37*	recessive
AHI1	*CA4*	dominant
AIPL1	*CC2D2A*	recessive
ALMS1	*CERKL*	recessive
ARL6	*CLRN1*	recessive
ARMS2	*CNGA1*	recessive
ATXN7	*CNGB1*	recessive
BBS1	*CRB1*	recessive
BBS10	*CRX*	dominant
BBS12	*DHDDS*	recessive
BBS2	*EYS*	recessive
BBS4	*FAM161A*	recessive
BBS5	*FLVCR1*	recessive
BBS7	*FSCN2*	dominant
BBS9	*GNPTG*	recessive
BEST1	*GUCA1B*	dominant
C1QTNF5	*IDH3B*	recessive
C2	*IMPDH1*	dominant
C2orf71	*IMPG2*	recessive
C3	*KLHL7*	dominant
C8orf37	*LRAT*	recessive
CA4	*MAK*	recessive
CABP4	*MERTK*	recessive
CACNA1F	*NR2E3*	recessive/dominant
CACNA2D4	*NRL*	recessive/dominant
CC2D2A	*OFD1*	X-linked
CDH23	*PDE6A*	recessive
CDH3	*PDE6B*	recessive
CDHR1	*PDE6G*	recessive
CEP164	*PRCD*	recessive
CEP290	*PROM1*	recessive
CERKL	*PRPF3*	dominant

CFB	*PRPF31*	dominant
CFH	*PRPF6*	dominant
CHM	*PRPF8*	dominant
CLN3	*PRPH2*	dominant
CLRN1	*RBP3*	recessive
CNGA1	*RDH12*	dominant
CNGA3	*RGR*	recessive
CNGB1	*RHO*	recessive/dominant
CNGB3	*RLBP1*	recessive
CNNM4	*ROM1*	dominant
COL11A1	*RP1*	recessive/dominant
COL2A1	*RP2*	X-linked
COL9A1	*RP8*	dominant
CRB1	*RP9*	dominant
CRX	*RPE65*	recessive/dominant
CYP4V2	*RPGR*	X-linked
DFNB31	*SAG*	recessive
DHDDS	*SEMA4A*	dominant
DMD	*SNRNP200*	dominant
EFEMP1	*SPATA7*	recessive
ELOVL4	*TOPORS*	dominant
ERCC6	*TTC8*	recessive
EYS	*TTPA*	recessive
FAM161A	*TULP1*	recessive
FBLN5	*USH2A*	recessive
FLVCR1		
FSCN2		
FZD4		
GNAT1		
GNAT2		
GNPTG		
GPR179		
GPR98		
GRK1		
GRM6		
GUCA1A		
GUCA1B		
GUCY2D		
HMCN1		

HTRA1		
IDH3B		
IFT140		
IMPDH1		
IMPG2		
INPP5E		
INVS		
IQCB1		
JAG1		
KCNJ13		
KCNV2		
KIF11		
KLHL7		
LCA5		
LRAT		
LRP5		
MAK		
MERTK		
MFN2		
MFRP		
MKKS		
MKS1		
MTTP		
MYO7A		
NDP		
NMNAT1		
NPHP1		
NPHP3		
NPHP4		
NR2E3		
NRL		
NYX		
OAT		
OFD1		
OPA1		
OPA3		
OPN1LW		
OPN1MW		
OPN1SW		

OTX2		
PANK2		
PAX2		
PCDH15		
PDE6A		
PDE6B		
PDE6C		
PDE6G		
PDZD7		
PEX1		
PEX2		
PEX7		
PGK1		
PHYH		
PITPNM3		
PLA2G5		
PRCD		
PRD		
PROM1		
PRPF3		
PRPF31		
PRPF6		
PRPF8		
PRPH2		
PXMP3		
RAX2		
RB1		
RBP3		
RBP4		
RCD1		
RD3		
RDH12		
RDH5		
RGR		
RGS9		
RGS9BP		
RHO		
RIMS1		
RLBP1		

ROM1		
RP1		
RP1L1		
RP2		
RP8		
RP9		
RPE65		
RPGR		
RPGRIP1		
RPGRIP1L		
RS1		
SAG		
SDCCAG8		
SEMA4A		
SLC24A1		
SNRNP200		
SPATA7		
TEAD1		
TIMM8A		
TIMP3		
TLR3		
TLR4		
TMEM126A		
TMEM237		
TOPORS		
TREX1		
TRIM32		
TRPM1		
TSPAN12		
TTC8		
TTPA		
TULP1		
UNC119		
USH1C		
USH1G		
USH2A		
VCAN		
WDPCP		
WDR19		

WFS1		
WDPCP		
WDR19		

Supplemental Table 2 Genes screened for 16 probands before NGS-based sequencing

Sample	Genes Screened
RC+V.27	*RP2, RPGR*
3H5+K.42	*IMPDH1, KLHL7, NR2E3, PRPF3, PRPF31, PRPF8, RDS, RHO, RP1, RP2*
3HV+M.66	*IMPDH1, KLHL7, NR2E3, PRPF3, PRPF31, PRPF8, RDS, RHO, RP1, RP2*
3WP+3.68	*IMPDH1, KLHL7, NR2E3, PRPF3, PRPF31, PRPF8, RDS, RHO, RP1, TOPORS*
3JY+V.17	*RP2, RPGR*
3JE+8.28	*RP2, RPGR*
5H3+9.73	*RP2, RPGR*
UY2+R.10	*CDH23, CLRN1, DFNB31, GPR98, MYO7A, PCDH15, USH1C, USH1G, USH2A*
5EF+L.58	*IMPDH1, KLHL7, NR2E3, PRPF3, PRPF31, PRPF8, RDS, RHO, RP1, TOPORS*
5WG+V.72	*IMPDH1, KLHL7, NR2E3, PRPF3, PRPF31, PRPF8, RDS, RHO, RP1, RP2*
LA+X.61	*RDS*
3W5+K.90	*IMPDH1, KLHL7, NR2E3, PRPF3, PRPF31, PRPF8, RDS, RHO, RP1, RP2*
375+8.59	*IMPDH1, KLHL7, NR2E3, PRPF3, PRPF31, PRPF8, RDS, RHO, RP1, RP2*
3VU+9.42	*IMPDH1, KLHL7, NR2E3, PRPF3, PRPF31, PRPF8, RDS, RHO, RP1, TOPORS*
3VF+9.12	*IMPDH1, KLHL7, NR2E3, PRPF3, PRPF31, PRPF8, RDS, RHO, RP1, TOPORS*
UPF+S.24	*ABCA4*

Figure Legends

Supplemental Figure S1. Electroretinogram results for patient UFC+7.74. Records shown here include Scotopic 0.01 (A), Scotopic 3.0 (B), Scotopic 3.0 Oscillatory Potentials (C), Photopic 3.0 (D), and Photopic Flicker [31 Hz] (E). Red lines represent right eye (OD) and blue lines represent left eye (OS). In all conditions, ERG responses were severely reduced or non-recordable.

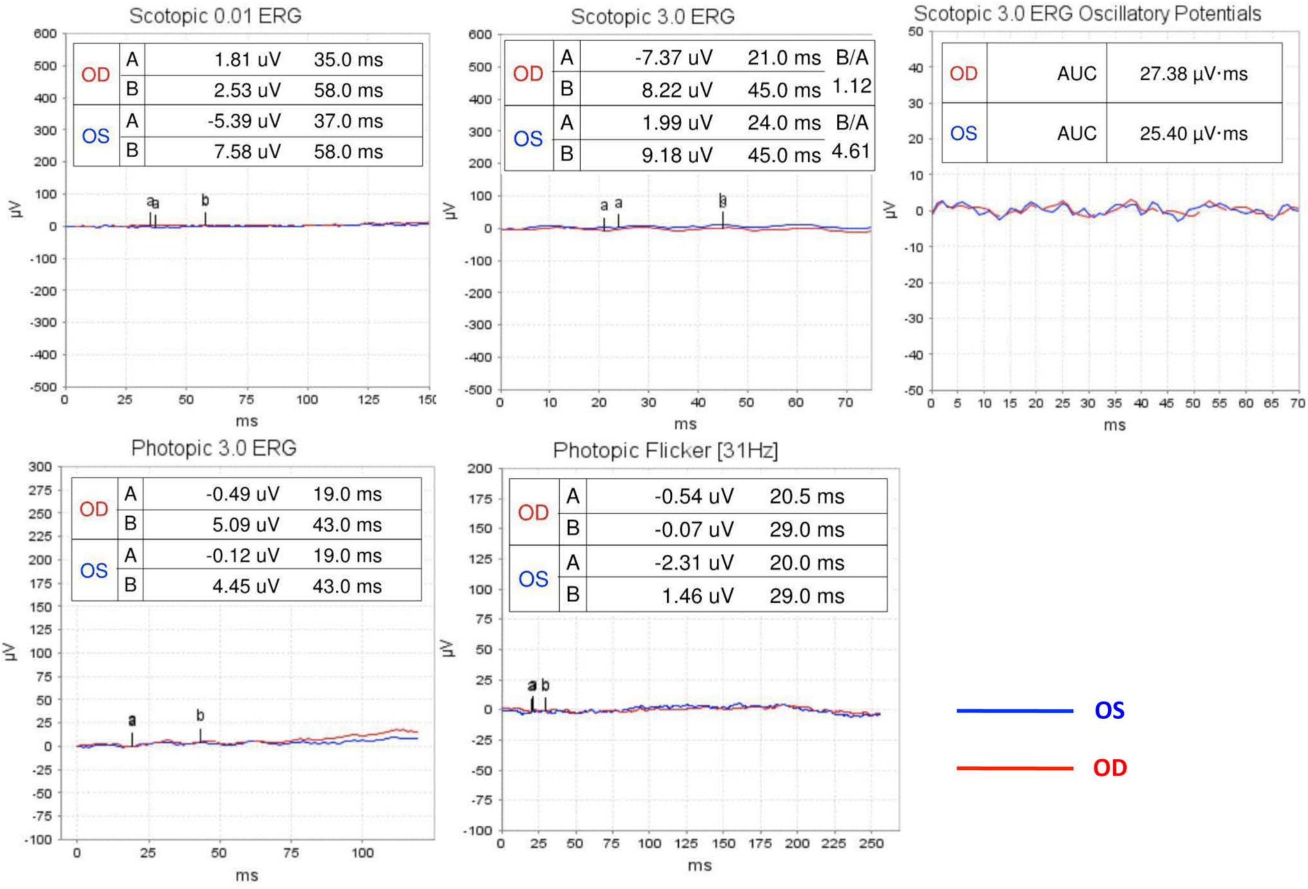

Scotopic 0.01 ERG
OD A 1.81 uV 35.0 ms
OD B 2.53 uV 58.0 ms
OS A -5.39 uV 37.0 ms
OS B 7.58 uV 58.0 ms
Scotopic 3.0 ERG
OD A -7.37 uV 21.0 ms B/A 1.12
OD B 8.22 uV 45.0 ms
OS A 1.99 uV 24.0 ms B/A 4.61
OS B 9.18 uV 45.0 ms
Scotopic 3.0 ERG Oscillatory Potentials
OD AUC 27.38 µV·ms
OS AUC 25.40 µV·ms
Photopic 3.0 ERG
OD A -0.49 uV 19.0 ms
OD B 5.09 uV 43.0 ms
OS A -0.12 uV 19.0 ms
OS B 4.45 uV 43.0 ms
Photopic Flicker [31Hz]
OD A -0.54 uV 20.5 ms
OD B -0.07 uV 29.0 ms
OS A -2.31 uV 20.0 ms
OS B 1.46 uV 29.0 ms
µV
ms
OS
OD
